# Modelling the Contributions of Malaria, HIV, Malnutrition and Rainfall to the Decline in Paediatric Invasive Non-typhoidal *Salmonella* Disease in Malawi

**DOI:** 10.1371/journal.pntd.0003979

**Published:** 2015-07-31

**Authors:** Nicholas A. Feasey, Dean Everett, E. Brian Faragher, Arantxa Roca-Feltrer, Arthur Kang’ombe, Brigitte Denis, Marko Kerac, Elizabeth Molyneux, Malcolm Molyneux, Andreas Jahn, Melita A. Gordon, Robert S. Heyderman

**Affiliations:** 1 Malawi-Liverpool-Wellcome Trust Clinical Research Programme, University of Malawi College of Medicine, Blantyre, Malawi; 2 Department of Clinical Sciences, Liverpool School of Tropical Medicine, Liverpool, United Kingdom; 3 Institute of Infection and Global Health, University of Liverpool, Liverpool, United Kingdom; 4 Leonard Cheshire Disability & Inclusive Development Centre, University College London, London, United Kingdom; 5 Department of Population Health, London School of Hygiene & Tropical Medicine, London, United Kingdom; 6 Department of Pediatrics, University of Malawi College of Medicine, Blantyre, Malawi; 7 Department of HIV & AIDS, Ministry of Health, Lilongwe, Malawi; 8 Division of Infection and Immunity, University College London, London, United Kingdom; Oxford University Clinical Research Unit, VIET NAM

## Abstract

**Introduction:**

Nontyphoidal Salmonellae (NTS) are responsible for a huge burden of bloodstream infection in Sub-Saharan African children. Recent reports of a decline in invasive NTS (iNTS) disease from Kenya and The Gambia have emphasised an association with malaria control. Following a similar decline in iNTS disease in Malawi, we have used 9 years of continuous longitudinal data to model the interrelationships between iNTS disease, malaria, HIV and malnutrition.

**Methods:**

Trends in monthly numbers of childhood iNTS disease presenting at Queen’s Hospital, Blantyre, Malawi from 2002 to 2010 were reviewed in the context of longitudinal monthly data describing malaria slide-positivity among paediatric febrile admissions, paediatric HIV prevalence, nutritional rehabilitation unit admissions and monthly rainfall over the same 9 years, using structural equation models (SEM).

**Results:**

Analysis of 3,105 iNTS episodes identified from 49,093 blood cultures, showed an 11.8% annual decline in iNTS (p < 0.001). SEM analysis produced a stable model with good fit, revealing direct and statistically significant seasonal effects of malaria and malnutrition on the prevalence of iNTS disease. When these data were smoothed to eliminate seasonal cyclic changes, these associations remained strong and there were additional significant effects of HIV prevalence.

**Conclusions:**

These data suggest that the overall decline in iNTS disease observed in Malawi is attributable to multiple public health interventions leading to reductions in malaria, HIV and acute malnutrition. Understanding the impacts of public health programmes on iNTS disease is essential to plan and evaluate interventions.

## Author Summary

Invasive nontyphoidal Salmonella (iNTS) disease is estimated to be responsible for 680,000 deaths/year and yet this is widely under-recognised by clinicians, epidemiologists and policymakers in Sub-Saharan Africa. Recently there have been reports of a decline in childhood iNTS disease from both Kenya and The Gambia that have been attributed to malaria control, and this has led to the unsubstantiated assumption that NTS disease will disappear. While this may be true in certain settings, numerous studies have also associated HIV and malnutrition with NTS disease. We therefore re-assessed this multifaceted relationship in Malawi, where we have previously reported little change in malaria cases, but where there have been highly successful antiretroviral and malnutrition programmes. Analysis of ~50,000 blood cultures and ~240,000 malaria slides demonstrates a significant decline in iNTS attributable to malaria, HIV and malnutrition, emphasising the complex inter-relationships between these factors and suggest that malaria interventions alone are unlikely to control iNTS disease. Our findings are highly relevant to the neglected field of bacteraemia in Africa and understanding the direct and indirect impacts of public health programmes on iNTS disease in Africa is essential for policy makers to plan and evaluate interventions to control this condition.

## Introduction

Blood stream infection (BSI) caused by non-typhoidal *Salmonella* (NTS) is consistently reported as a major cause of morbidity and mortality in children across sub-Saharan Africa (SSA), especially those aged between 6 and 30 months.[1] As pathogens such as *Haemophilus influenzae* type b (Hib), *Neisseria meningitidis* serogroup A, and *Streptococcus pneumoniae* are targeted by highly effective protein-conjugate vaccines, life-threatening disease caused by invasive NTS (iNTS) is likely to become relatively more prominent [2].

The strong epidemiological association between malaria and iNTS disease has been documented in several African countries, including Malawi [1, 3, 4], and there is increasing biological evidence that multiple malaria-induced immune defects predispose to iNTS disease, including iron release from haem, impaired neutrophil function and reduction in IL12 production [5–7]. In addition, there are many other factors which may influence host susceptibility to invasive NTS disease among children, including inadequate protective antibody [8, 9], malnutrition [10] and impaired cell-mediated immunity caused by HIV infection [11–13]. Recently, a temporal association between a decline in the incidence of malaria and a falling incidence of iNTS disease has been reported from both The Gambia and Kenya [14, 15]. This has led to the suggestion that effective population-based malaria interventions might result in control of iNTS disease across the continent without the need for specific NTS-targeted measures.

In Malawi, we have observed a fall in iNTS disease over 10 years of bacteraemia sentinel surveillance. Here, large-scale implementation of malaria control interventions gained considerable momentum in 2007; however, by 2010 these interventions had yet to impact on the incidence of mild and severe malaria [16]. Alongside this, there has been a highly effective roll-out of antiretroviral therapy (ART) since 2004, with scale up of prevention of mother to child transmission (PMTCT) from 2006 [17, 18]. In addition, a programme to subsidize fertilizer for subsistence farmers, which began in 2005 [19], has contributed to reductions in all measures of child malnutrition between 2004 and 2010 [20]. Finally, we have previously described a strong seasonal relationship between rainfall and iNTS disease [21]. We hypothesised that the underlying risk factors of rainfall, malnutrition and HIV, in addition to malaria, would be associated, directly and/or indirectly, with the observed changes in iNTS disease incidence.

Many of these risk factors are known to interact. To assess the complex interrelationships of factors associated with iNTS disease, we have used structural equation modelling (SEM) to analyse longstanding surveillance data collected at the largest government hospital in Malawi from 2001–2010. The inter-relationships between the monthly numbers of malaria, malnutrition and HIV cases and their association with the corresponding monthly numbers of iNTS cases presenting over the same time period have been modelled in the context of rainfall levels which potentially have both direct environmental effects on NTS transmission through increased surface water and indirect effects on host susceptibility largely through malaria transmission and under-nutrition in the rainy season.

## Methods

### Study site and population

Queen Elizabeth Central Hospital (QECH) is a 1250-bed government funded hospital, serving a population of approximately 1 million. Approximately 50,000 children/year are assessed at QECH of whom approximately a quarter are admitted. Malaria transmission is endemic, with seasonal peaks during the rainy season [22]. Admissions for severe acute malnutrition (SAM) also peak during the rainy season, as increased infection risk coincides with the peak of the ‘hungry season’; when household food supplies are running low whilst the new season’s crops are growing. The prevalence of HIV in pregnant women was approximately 22% within Blantyre in 2001, declining to 16% by 2010 (Malawi Ministry of Health Quarterly HIV Programme Reports). Vertical transmission of HIV was estimated to occur in 17% of HIV infected pregnancies prior to 2008 and had declined to 13.5% by 2010 [23]. In Blantyre, 89% of HIV infected children died by the age of 3 years prior to the roll-out of ART [24].

### Clinical management and laboratory methods

The primary route of admission for children is the pediatric Accident and Emergency (A&E) Unit. All children presenting unwell to hospital with non-surgical illness have blood obtained for a thick blood-film malaria parasite examination, and, if slide-positive were diagnosed with malaria; no account was taken of severity in this study.

Blood cultures are obtained from children in whom sepsis is suspected and the criteria for obtaining blood for culture did not change during the study period, nor did the numbers of cultures taken [25]. Automated blood culture was undertaken using a pediatric bottle (BacT/Alert PF; BioMerieux, UK) incubated at 37°C in air. Gram-negative isolates were identified using standard techniques; including over-night incubation on blood and MacConkey agar at 37°C in air, and if oxidase negative, identified by API 20E (BioMerieux, UK). Salmonellae were then serotyped as *S*. Enteritidis, *S*. Typhimurium, *S*. Typhi or *S*. sp. by the following antisera; polyvalent O & H, O4, O9, Hd, Hg, Hi, Hm, and Vi (Prolab Diagnostics, UK)[26].

### Data abstraction

Numbers of *P*. *falciparum-*positive blood-slides are recorded at the end of each month at the paediatric A&E unit at QECH and an analysis of trends of slide-positivity and of severe disease, from January 2001 to December 2010, has previously been undertaken [16]. To relate trends of malaria infection to indices of iNTS disease, all blood cultures collected from paediatric admissions during the same period were reviewed. Daily rainfall data (mm) were obtained for the Blantyre District from the Department of Climate Change and Meteorological Services, Malawi. As national nutritional data were only available for two time points, monthly admission numbers to the ‘Moyo’ Nutritional Rehabilitation Unit (NRU) at QECH were used as a proxy indicator of the incidence of SAM. The admission policy for the NRU did not change throughout the study period, following standard definitions of SAM: weight for height < 70% of the NCHS reference median and/or nutritional oedema, and/or a mid-upper arm circumference (MUAC) < 110 mm [27]. Admissions to the NRU almost all come through the paediatric A&E. Only rarely, if children are very sick or their malnutrition is initially missed, are they first admitted to the general wards and transferred later.

### Data estimates

As data were unavailable for malaria for the fourth quarter of 2004 and NRU admissions data were unavailable for 2005, these data had to be estimated. As both variables are strongly seasonal, estimates were made for each missing month by calculating the mean of the corresponding month one year before and one year after.

As there is evidence that risk of iNTS disease due to HIV-infection declines following effective ART, and that the vast majority of cases of paediatric iNTS disease occur in children under 3 years of age, an estimate was made for the number of children under 3 years in Blantyre with untreated HIV during the study period [28–30]. This estimate was made by taking the estimated number of HIV-infected pregnancies per year in Blantyre during the study period and multiplying this by the estimated incidence of vertical transmission, assuming a 1%/year fall from 18% in 2006 to 14% in 2010 (Government of Malawi, Ministry of Health: Quarterly HIV Program Reports (2005–2014) https://www.hiv.health.gov.mw/index.php/our-documents) [23]. Malawi Ministry of Health ART programme data were used to estimate the number of children in Blantyre on effective ART, based on the programme starting in 2006 and reaching 30% coverage by 2010, and estimating that ART achieved effective protection against iNTS disease in 70% of recipients. Mortality was estimated at 30%/year in the first three years of life for those not on ART, based on published studies from Malawi (S1 Table)[24].

### Statistical methods

**Fig 1:**
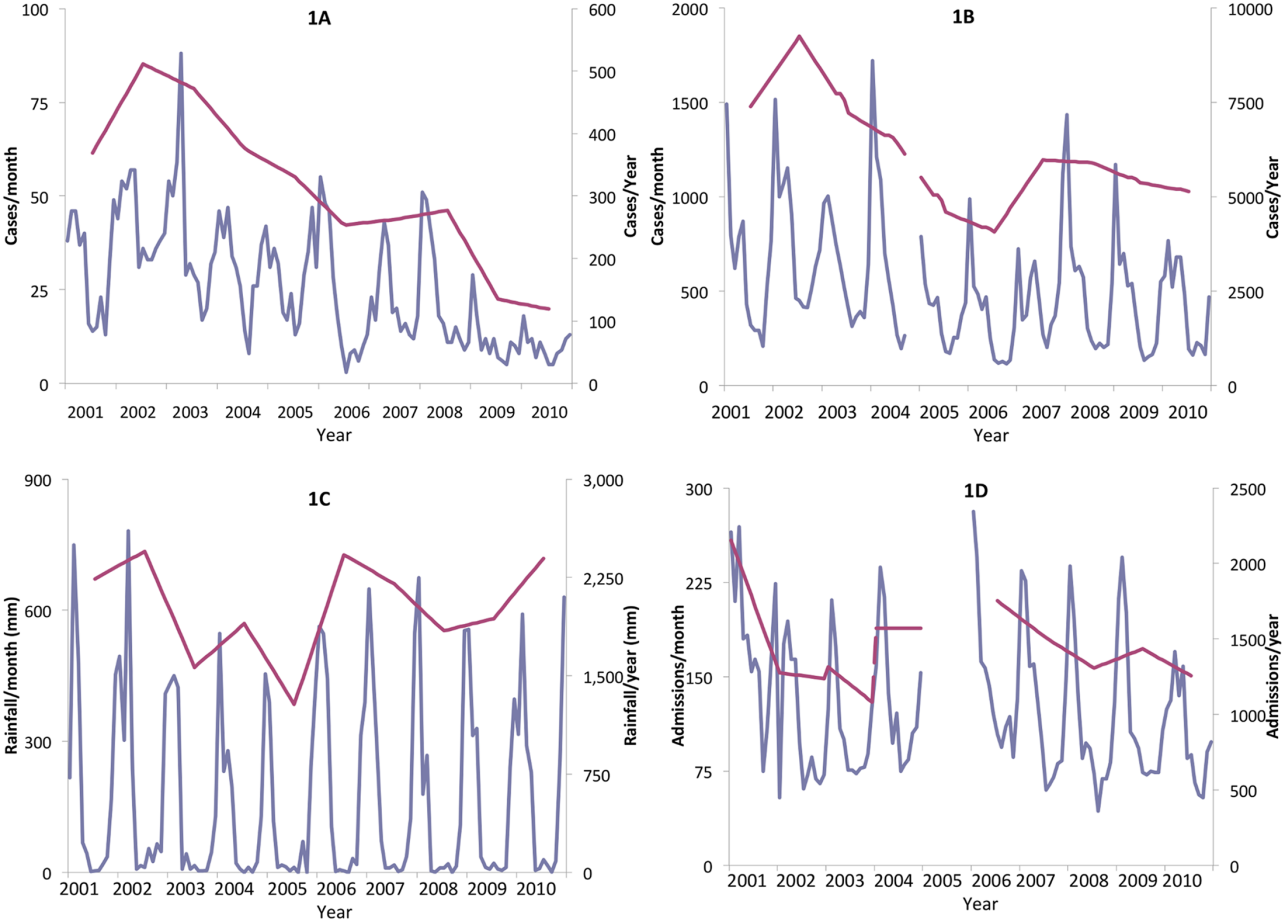
Trends in paediatric iNTS disease numbers (1A). Paediatric malaria case numbers (1B), rainfall in mm (1C) and admissions to the Nutritional Rehabilitation Unit (1D). The Blue line (left axis) is by month and the red line (right axis) by year.

The total numbers of iNTS cases, malaria cases and admissions to the NRU were computed, along with the total rainfall (in mms), for each month of the study period (Fig 1). As the predictor variables (rainfall, malnutrition, HIV and malaria prevalence) were inter-related, a series of structural equation models (SEM) were fitted to the data [31]. SEM allows a much more complex set of hypothesised inter-relationships to be explored between variables than is possible with standard multivariable linear regression methods. The latter requires the assumption that a set of predictor variables are independently associated with (usually a single) outcome variable. For the specific context of the objectives of this study, SEM methods construct a Bayesian network comprising nodes, representing the variables selected for investigation, linked by arrows indicating probabilistic relationships. A standardised regression coefficient for each line, calculated by the software, indicates the relative contribution of each relationship. This network allows the model to describe additional relationships between the multiple predictor variables. In addition, since the probability and regression coefficient represented by arrows in the model varies according to the direction of the arrow, SEM methods provide stronger evidence for interpreting variable parameters as causal effects, when analysing data from cross-sectional studies, than is the case with multivariable linear regression models. [32].

A simple graphical examination of the incidence estimates for iNTS, malaria, malnutrition and rainfall showed the expected strong monthly cyclical seasonal patterns along with year-on-year reductions, while HIV prevalence did not show a seasonal pattern (Fig 1). In the first SEM, the monthly numbers of iNTS cases, malaria cases, HIV cases, NRU admissions and rainfall levels were analysed in order to model cyclical monthly variations in these variables. In the second SEM, however, the data were smoothed by taking a 12-month rolling means in order to remove the effect of month-by-month seasonality and hence evaluate the impact of variables acting on iNTS disease over longer periods, in particular HIV-infection. Goodness-of-fit of each SEM was determined using a chi-square test and the root mean square error of approximation (RMSEA) statistic [33]. All SEMs were constructed using the IBM SPSS Amos (release 20.0) software package. For this exploratory model, statistical significance was set at an alpha of 10%.

## Results

A total of 49,093 blood cultures were taken between January 2001 and December 2010. Of these, 21% (n = 10,265) yielded isolates of clinical significance and 7,244 (15%) yielded likely contaminants. NTS were isolated from 3,105 (30%) of 10,265 significant blood cultures (Fig 1). The iNTS disease epidemic peaked in 2002. Monthly malaria diagnostic data from the paediatric A&E unit were available for the same period, except from October to December 2004. A total of 242,953 slides were taken for malaria between January 2001 and December 2010 and 61,320 (25.2%) of these were found to be *P*. *falciparum* positive.

Totalled by year (Table 1 and Fig 1), iNTS incidence fell on average by 11.8% annually (p<0.001), while malaria incidence fell on average by 4.0% per year (p < 0.001) largely due to a fall between 2001 and 2003; admissions to the NRU fell on average by 5.8% per year (p = 0.143). There were marked variations in the average rainfall levels over the study decade but no evidence of any systematic trend, either upwards or downwards (p = 0.927).

**Table 1:** Total invasive Nontyphoidal Salmonella (iNTS) disease cases, malaria cases, annual rainfall and nutritional rehabilitation unit (NRU) admissions by year.

	Year									
	2001	2002	2003	2004	2005	2006	2007	2008	2009	2010
**iNTS cases**	369	511	472	376	330	254	263	277	135	119
**Malaria cases**	7392	9251	7217	6429*	4591	4062	5969	5903	5374	5132
**Total rainfall (mms)**	2240	2449	1565	1897	1282	2424	2202	1843	1935	2397
**Admissions to NRU**	2154	1275	1315	1571	---	1752	1508	1307	1434	1255

*Malaria cases not recorded for Oct, Nov and Dec 2004

### Structural equation modelling

**Fig 2:**
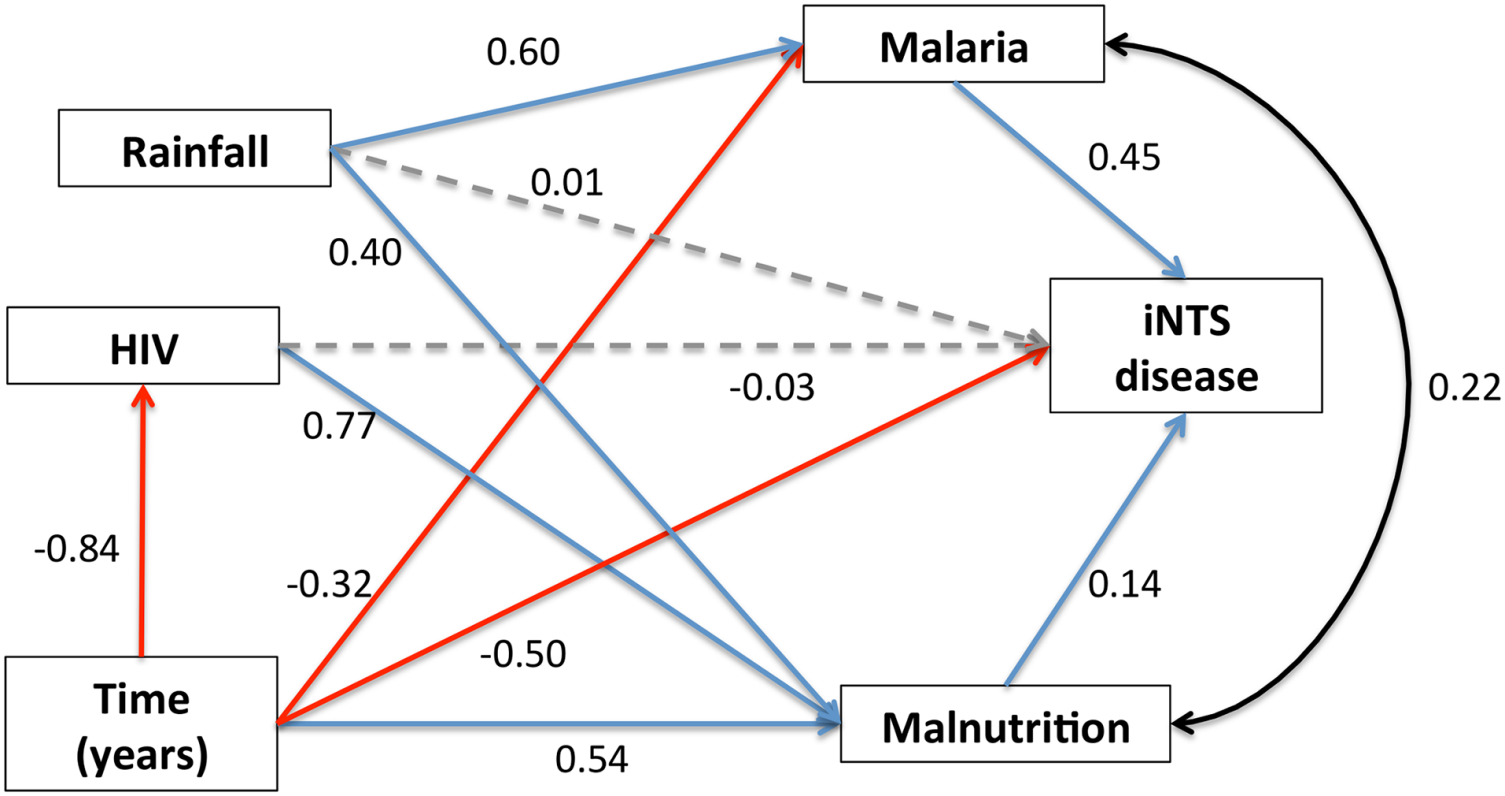
Seasonal structural equation model (SEM) of the interaction between malaria, malnutrition, HIV, rainfall, and time upon iNTS disease. Numbers are standardised regression coefficients from SEM model fits. Blue lines indicate statistically significant *positive* relationships; red lines indicate statistically significant *negative* relationships; grey lines indicate statistically *non-*significant relationships. The black line indicates a non-directional correlation.

**Fig 3:**
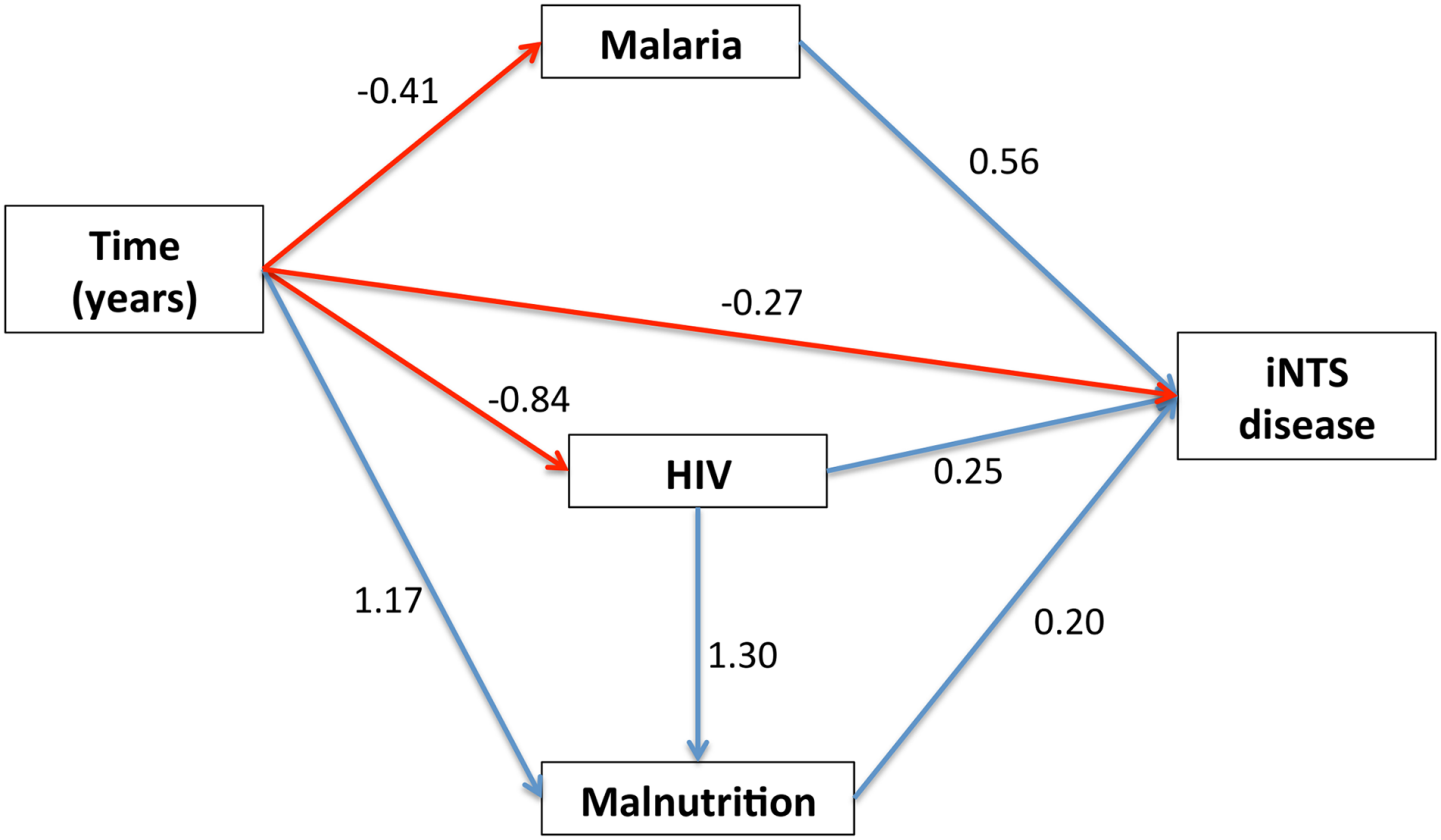
Structural equation model (SEM) of the interaction between malaria, malnutrition, HIV and time upon iNTS disease using smoothed data. Seasonality has been smoothed using a 12-month rolling average, allowing the impact of longer-term trends to be examined. Numbers are standardised regression coefficients from SEM model fits. Blue lines indicate statistically significant *positive* relationships; red lines indicate statistically significant *negative* relationships; grey lines indicate statistically *non-*significant relationships. The black line indicates a correlation.

We hypothesised that the control of multiple conditions associated with increased risk of iNTS disease, including malaria, HIV and malnutrition, was likely to have led to a decline in iNTS disease. SEM were therefore constructed from the peak in of the epidemic in 2002 to 2010 (Fig 2 and Fig 3 and S1 Table for complete monthly data). The relationships between variables, including seasonal variations, were explored by modelling monthly data describing culture-confirmed iNTS disease, slide-positive malaria cases, NRU admissions (representing malnutrition), rainfall and HIV (Fig 2). This demonstrated statistically significant and direct contributions to iNTS disease from both malaria and malnutrition. There was also a non-directional correlation between malaria and malnutrition. In this model rainfall had no direct impact upon iNTS disease, but had a strong and significant impact on iNTS disease through its effects upon malaria and also upon malnutrition, lending biological plausibility to the model. This model suggested that whilst HIV had no direct effect on iNTS disease, it indirectly contributed to iNTS disease through its effect upon malnutrition. Time was found to have statistically significant negative relationships with some variables, suggesting that other factors outwith the model were contributing to the marked decline of malaria and HIV disease in Blantyre, as indicated by high standardised regression coefficients. The smaller negative effect of time upon iNTS suggests there were also other factors out-with the model contributing to the fall in iNTS, which we did not capture with our data. This model has good statistical strength (chi-square(3) = 4.423, p = 0.219; RMSEA = 0.067 (90% CI: <0.001–0.188).

In order to investigate long-term non-seasonal trends in iNTS disease, a second SEM was constructed, this time smoothing seasonality out of the data (iNTS disease, malaria and malnutrition) by taking a 12-month rolling mean (Fig 3). This enabled the effect of HIV prevalence, which is not seasonal and which changes over longer time periods, to be better evaluated. Rainfall did not contribute any statistically significant relationships once month-to-month seasonality was removed, and was therefore not included in this model. As is the case in the first model, both malaria and malnutrition continued to exhibit major direct relationships with iNTS disease, and the previous non-directional correlation also disappeared. In this model, however, HIV also demonstrated a direct, significant effect upon iNTS disease in addition to an indirect one through its contribution to malnutrition. Similar time effects on iNTS disease, malaria and HIV were seen in this model. In this model, the standardised regression coefficient for the association between time and numbers of iNTS cases presenting was approximately 50% of the corresponding coefficient for the first model, suggesting that there were fewer unexplained factors in this model acting on the observed decline in iNTS cases. Once again, in addition to the statistically significant interactions of the variables within the model, the overall model fit was good (chi-square(2) = 1.121, p = 0.571; RMSEA < = 0.001 (90% CI: <0.001–0.162).

## Discussion

In many populations in SSA, we are largely ignorant of the burden of iNTS disease, impacts of potential risk factors, routes of transmission, and long-term trends [2]. Without such knowledge, it is impossible to plan and evaluate interventions. Analysis of very large data sets from Blantyre, Malawi reveals a significant and sustained decline in iNTS disease, following an epidemic peak between 2001–2004. There has not been a comparable or sustained decline in the malaria slide positivity rate since that described between 2001 and 2003 [16], a finding which is corroborated across Malawi by WHO surveillance [34]. Whilst our data are from a single hospital setting and might have failed to appreciate a decline in mild malaria in the community, both admissions with cerebral malaria, and asymptomatic parasitaemia on an orthopaedic unit, reflected clinical malaria cases throughout the study period [16]. We therefore hypothesized that the decline in paediatric malaria cases from a peak in 2001 to the beginning of a plateau in 2003 had contributed to the initial observed fall in iNTS, but that subsequently, other factors such as the prevalence of HIV and malnutrition were also important contributors.

We used SEM analytical methods to unravel this complex relationship, as the factors associated with iNTS disease are interrelated and cannot be addressed using standard regression methods. This allows the possibility of exploring an indirect association between a predictor variable and the outcome variable through additional predictor variables, therefore allowing for mediating and confounding effects to be modelled [31].

Malaria undoubtedly contributed significantly and directly to the decline in iNTS disease, consistent with the experiences reported in the Gambia and Kenya [14, 15], and was the variable that was most strongly associated with iNTS disease in both models. Our exploratory modelling, however, clearly demonstrated that other factors are associated with reductions in iNTS disease, whether by direct or indirect mechanisms or both. It is clear that HIV and malnutrition contribute to changes in the incidence of iNTS disease, through both direct and indirect effects. Seasonal rainfall, by contrast, exerted a complex and important indirect effect on iNTS disease through its contribution to both malaria and malnutrition.

HIV was previously associated with 18% of childhood iNTS disease in a study of Kenyan children [10]. Several developments in Malawi have reduced the prevalence of HIV infection among young children in Blantyre, including extensive anti-retroviral therapy (ART) roll-out since 2005 and a national program of peri-partum ART to reduce mother-to-child transmission of HIV, and we hypothesised that this has had an impact on iNTS disease [23]. In the SEM displaying seasonal variation, HIV prevalence in Blantyre indirectly impacted upon iNTS through its effect upon malnutrition, highlighting the strength of this modelling methodology. When the effect of month-on-month seasonality was smoothed out to account for the lack of seasonality of HIV, it retained a very strong influence on malnutrition, and had an additional direct, significant effect upon iNTS disease. This model suggested that the combined effects of HIV and malnutrition were of similar magnitude to that of malaria on iNTS incidence.

The prevalence of underweight-for-height status in children aged <5 years in Malawi, which was static between 2000–2004, has declined from 6% in 2004 [35] to 4% in 2010 [20], and this reduction was reflected in the decline in NRU admissions to QECH. SEM using NRU admissions revealed that nutrition has strong interactions with both rainfall and HIV. The smoothed model suggested that approximately half NRU admissions were explained by variations in HIV-prevalence. Of note, the positive standardised regression from year to NRU admissions, suggests that NRU admissions are gradually increasing, once reductions due to other factors in the model such as HIV and iNTS disease are taken into account, emphasising the complex and multi-causal epidemiology of malnutrition.

In contrast, the model also reflects the very significant reductions over time of both malaria and HIV once factors outwith the model are accounted for, a finding that we interpreted as reflecting the known successes of recent control programmes for malaria and HIV in Malawi.

### Limitations

These observational data come from a single centre within Blantyre, and it is likely that some young children die from unidentified and untreated iNTS in the community. Furthermore, we do not have a longitudinal survey reflecting changes in patterns of health service utilisation from this surveillance period. Other factors such as changing availability of medications such as antibiotics or anti-malarials might also have exerted an unseen or proxy effect on our data. Our use of NRU admissions as a proxy indicator for population nutrition status means that nutrition-related observations must be interpreted with care as the NRU only admits cases of SAM. These do not necessarily reflect population distributions (particularly after mid 2008 when community-based SAM treatment began in Blantyre district) or the impact of the two other forms of malnutrition, underweight and stunting. Nonetheless, they do reflect the national data relating to all forms of under-nutrition—and as the models presented use actual hospital admission figures, they accurately measure disease burden on health facilities in the study area.

Whilst our models have good statistical strength, the changes in iNTS disease that were directly attributed to time indicate that factors outwith the model also affect iNTS disease in Malawi. In particular, it is likely that access to drinkable water and to sanitation and hygiene (WASH) facilities affect iNTS disease and we have not been able to include data reflecting changes in provision of WASH facilities in Blantyre over the study period in the model.

### Conclusions

Our data suggest that the interaction between iNTS disease and malaria, HIV and malnutrition is complex, and that the observed decline in iNTS disease is likely to have been due to multiple public health interventions. We estimate that slightly less than half of this change is explained by a decline in malaria and that a similar proportion was explained by changes in the local epidemiology of HIV, both directly and through its impact on malnutrition. We illustrate the potential of modelling methodology and sentinel surveillance data to inform the use of public health interventions to reduce iNTS disease in SSA. The model indicates some gaps in the data, suggesting that there are other unknown factors that also appear to influence the incidence of iNTS disease. It is therefore likely that direct interventions such as NTS vaccines or improvements in WASH facilities will be required to match recent achievements seen in the control of other severe life-threatening bacterial infections such as *Haemophilus influenzae* Group b, pneumococcal and meningococcal disease.

## Supporting Information

S1 Table Complete monthly data used for structural equation modelling. (XLSX)

S1 Checklist STROBE checklist. (PDF)
